# Overexpression of *Mitochondrial Phosphate Transporter 3* Severely Hampers Plant Development through Regulating Mitochondrial Function in *Arabidopsis*

**DOI:** 10.1371/journal.pone.0129717

**Published:** 2015-06-15

**Authors:** Fengjuan Jia, Xiaomin Wan, Wei Zhu, Dan Sun, Chengchao Zheng, Pei Liu, Jinguang Huang

**Affiliations:** 1 State Key Laboratory of Crop Biology, College of Life Sciences, Shandong Agricultural University, Taian, Shandong, P.R. China; 2 College of Food Science and Engineering, Shandong Agricultural University, Taian, Shandong, P.R. China; Universidade Federal de Vicosa, BRAZIL

## Abstract

Mitochondria are abundant and important organelles present in nearly all eukaryotic cells, which maintain metabolic communication with the cytosol through mitochondrial carriers. The mitochondrial membrane localized phosphate transporter (MPT) plays vital roles in diverse development and signaling processes, especially the ATP biosynthesis. Among the three *MPT* genes in *Arabidopsis* genome, *AtMPT3* was proven to be a major member, and its overexpression gave rise to multiple developmental defects including curly leaves with deep color, dwarfed stature, and reduced fertility. Transcript profiles revealed that genes involved in plant metabolism, cellular redox homeostasis, alternative respiration pathway, and leaf and flower development were obviously altered in *AtMPT3* overexpression (OEMPT3) plants. Moreover, OEMPT3 plants also accumulated higher ATP content, faster respiration rate and more reactive oxygen species (ROS) than wild type plants. Overall, our studies showed that *AtMPT3* was indispensable for *Arabidopsis* normal growth and development, and provided new sights to investigate its possible regulation mechanisms.

## Introduction

Mitochondria are important organelles that can integrate numerous metabolic pathways, including energy homeostasis, redox balance, cellular growth and stress adaptation [1, 2]. The drastic expression fluctuation of mitochondrion associated genes would disturb mitochondrial function, which had significant effects on growth and development. For example, overexpression of the unedited form of the subunit 9 of ATP synthase gene (*35S*::*u-atp9*) caused mitochondrial dysfunction, dwarf morphology and male sterility [3]. Loss of the mitochondrial Mg^2+^ transporter *AtMGT5* led to pollen abortion and male sterility [4]. Transgenic *Arabidopsis* plants overexpressing mtDNA-asscciated *AtWhy2* showed defects in mitochondrial function, smaller and distorted leaves [5]. Double mutant of mitochondrial malate dehydrogenase (mMDH) *mmdh1mmdh2* showed growth retardation due to the lower net CO_2_ assimilation rate [6]. All these studies indicate that finely tuned mitochondrion activities are indispensable for plant normal growth and development.

The transport of various metabolites, nucleotides and cofactors across the inner mitochondrial membrane relies on the presence of a family of related proteins: the mitochondrial carrier family. This superfamily contains a tripartite structure of 100 amino acid segments each consisting of two membrane-spanning α-helices separated by an extra-membrane hydrophilic loop [7]. These transporters operate as homodimers with a 12 transmembrane domain structure and are responsible for the transport of a wide variety of metabolites between mitochondrion and cytosol [8]. In *Arabidopsis*, the mitochondrial carrier family contains as many as 58 members [9]. In recent years, several of these transporters have been cloned and investigated at the molecular and biochemical levels, including the adenine nucleotide transporter (ANT), the oxoglutarate-malate transporter (OMT), the uncoupling protein (UCP), the dicarboxylate transporter, the tricarboxylate transporter and the phosphate transporter [10–14]. However, the biological functions of most members in this family remain unknown. In eukaryotes, the mitochondrial phosphate transporters (MPTs), which belong to the mitochondrial carrier family, play crucial roles in respiration by uptaking orthophosphate (Pi) into the mitochondrial matrix, where the Pi is utilized for the conversion of ADP to ATP [15–17]. In human, disorder of the MPT coding gene *SLC25A3*, which was essential for ATP biosynthesis, could lead to lactic acidosis, hypertrophic cardiomyopathy and muscular hypotonia [18]. Since the first isolation of plant *MPT* gene from birch [16], numerous *MPT* homologues have been cloned and characterized in other plant species, including soybean, maize, rice, *Arabidopsis*, *Lotus japonicus* and *Paeonia suffruticosa* [15, 19, 20]. In *Arabidopsis*, there are three *MPT*s, including *AtMPT1* (AT2G17270), *AtMPT2* (AT3G48850) and *AtMPT3* (AT5G14040), encoding protein with 309, 363 and 375 amino acids, respectively [20]. Among them, *AtMPT2* and *AtMPT3* have been proved to have phosphate transport activity [17]. In our previous work, *AtMPTs* were shown to be involved in salt stress tolerance [21]. Intriguingly, overexpression of *AtMPT3* also led to severe developmental defects. However, the underlying molecular mechanisms between *AtMPT3* and developmental defects were still unknown.

Reactive oxygen species (ROS), including singlet oxygen, hydroxyl radical, superoxide anion radical and hydrogen peroxide, are constantly produced in plants and accumulate under stressful conditions [22, 23]. Although ROS have been identified as important signaling molecules in diverse biological processes, its excessive production was toxic [24]. *Arabidopsis* plants lacking ubiquitin-specific protease 16 displayed significant increase in H_2_O_2_ accumulation and severe cell death [25]. Deficiency of *Arabidopsis* frataxin altered the activity of mitochondrial Fe–S proteins and exhibited increased formation of ROS, retarded plant growth, reduced fresh weight and seed number [26]. The mutation in *SLG1* (*Slow Growth 1*) disrupted the function of mitochondrial complex I and accumulated large amount of H_2_O_2_, and exhibited lower growth rates [27]. These observations also raised an interesting biological question about the relationships between ROS accumulation and plant development.

In this paper, we reported that *Arabidopsis* overexpressing *AtMPT3* displayed multiple morphological phenotypes, including deformed leaves, dwarfed stature and reduced fertility. Microarray analysis revealed that the enhanced expression of *AtMPT3* altered the transcript levels of numerous genes involved in plant metabolism, cellular redox homeostasis, alternative respiration pathway, and leaf and flower development. Furthermore, a much higher ATP content, respiration rate and ROS level were also detected in the transgenic plants. These results suggest that *AtMPT3* had important effects on plant growth and development via regulating the mitochondrial function.

## Materials and Methods

### Plant materials and growth conditions

Seeds of *Arabidopsis* (ecotype Columbia) were surface sterilized, and plated on 1/2 Murashige and Skoog (MS) medium [28]. The plates were kept in the dark at 4°C for 3 days and then transferred to a growth chamber with a light/dark cycle of 16-h-light/8-h-dark at 22°C for 7 days. Then seedlings were transferred to growth chamber and grown at 22°C under 8-h-light/16-h-dark (for vegetative growth) or 16-h-light/8-h-dark (for reproductive growth).

### Vector construction and transgenic plant generation

The coding region of *AtMPT3* was cloned into the pBI121 binary vector under the control of CaMV 35S promoter. The constructs were introduced into *Agrobacteriun tumefaciens* strain GV3101 and then transformed into *Arabidopsis* by floral dip method [29].

### RNA extraction and real time RT-PCR analysis

Total RNA from wild type and transgenic plants was extracted using a universal plant total RNA extraction kit (BioTeke, China). Contaminated DNA was removed with RNase-free DNase I. cDNA was synthesized using PrimeScript RT (reverse transcriptase) with oligo-dT primer using the PrimeScript RT master mix kit (Takara, Japan). A SYBR green real-time PCR master mix (Takara, Japan) and a Chromo 4 real-time PCR detector (Bio-Rad, USA) were used. Three biological replicates and three technical replicates were performed for the real time RT-PCR experiment with *GAPDH* as an internal control.

### Microarray experiments and data analysis

RNA was labeled with the Message-Amp II-Biotin Enhanced kit. After verifying RNA integrity by the Agilent RNA 6000 Nano Kit and the Agilent 2100 Bioanalyzer, the labeled RNA was hybridized to Affymetrix ATH1 Genome arrays at ATLAS Biolabs. Hybridizations were done in three biological replicates. The data were analyzed using Robin [30] with the default settings of RMA (robust multi array averaging) with *P* < 0.05, and visualized by MapMan [31] and PageMan [32].

### Histochemical staining

For H_2_O_2_ and O_2_^-^ detection, plants were fixed with DAB (3’, 3’-diaminobenzidine) and NBT (nitro blue tetrazolium) as described by Orozco-Cardenas [33] and Kawai-Yamada [34], respectively. To quantify formazan generation, stained samples were boiled in dimethyl sulfoxide until formazan precipitates were eluted completely [35]. The amount of formazan was determined using spectrophotometer at 560 nm. Trypan blue staining was performed as previously described [36]. Pollen from newly dehiscing flowers was deposited, and the vitality stain was accomplished on pollen as described by Alexander [37]. GUS staining was carried out according to Zhu *et al*.[21].

### Measurement of anthocyanin, ATP and respiration rate

Anthocyanin quantification was performed as described by Deikman and Hammer [38]. Two absorbances (A_535_ and A_650_) of the extracts were measured using spectrophotometer. The amount of anthocyanins was reported as (A_535_—A_650_) g^-1^ FW (fresh weight). The ATP concentration was measured as described previously [39]. Briefly, 250 mg of 20-day-old leaf were ground and resuspended in 400 mL of 2.3% (v/v) trichloroacetic acid. A bioluminescent assay kit (Sigma-Aldrich) and an ultraviolet-visible spectrophotometer was used to measure the ATP concentration. The respiration rate of 20-day-old rosette leaf was measured using a Clark-type oxygen electrode as described previously [40]. Three biological repeats were carried out for both the ATP content and the respiration rate assays.

## Results

### Overexpression of *AtMPT3* hampers the growth and development in *Arabidopsis*

To understand the biological functions of *AtMPT3*, transgenic *Arabidopsis* constitutively expressing *AtMPT3* cDNA fused with 35S promoter were generated. We obtained 17 independent transgenic lines. To determine the expression levels of *AtMPT3* in these lines, total RNA from 2-week-old wild type and transgenic plants were extracted and analyzed by real time RT-PCR, and line5 (L5) showed the highest expression level (S1 Fig). Then, L5 (also termed OEMPT3) and two other transgenic lines (L4 and L14) were selected for further analysis.

**Fig 1:**
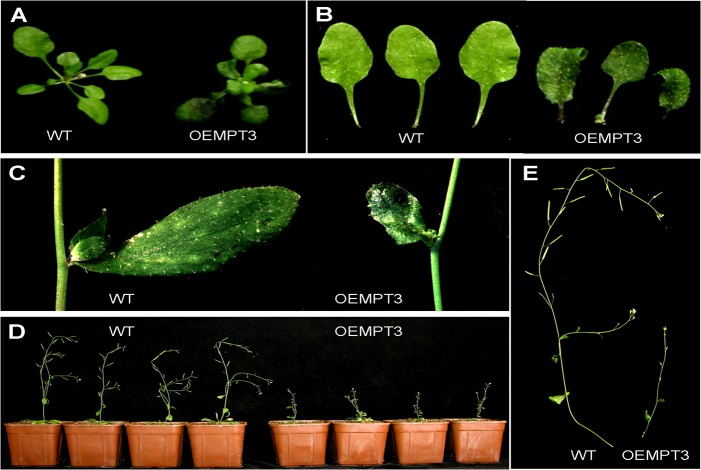
Developmental phenotypes of OEMPT3 plants at seedling stage. (A, B) The rosette leaves of wild type and OEMPT3 plants at 20 DAP. (C) The cauline leaves of wild type and OEMPT3 plants at 40 DAP. (D, E) Phenotypes of OEMPT3 plants compared to wild type at 60 DAP.

**Fig 2:**
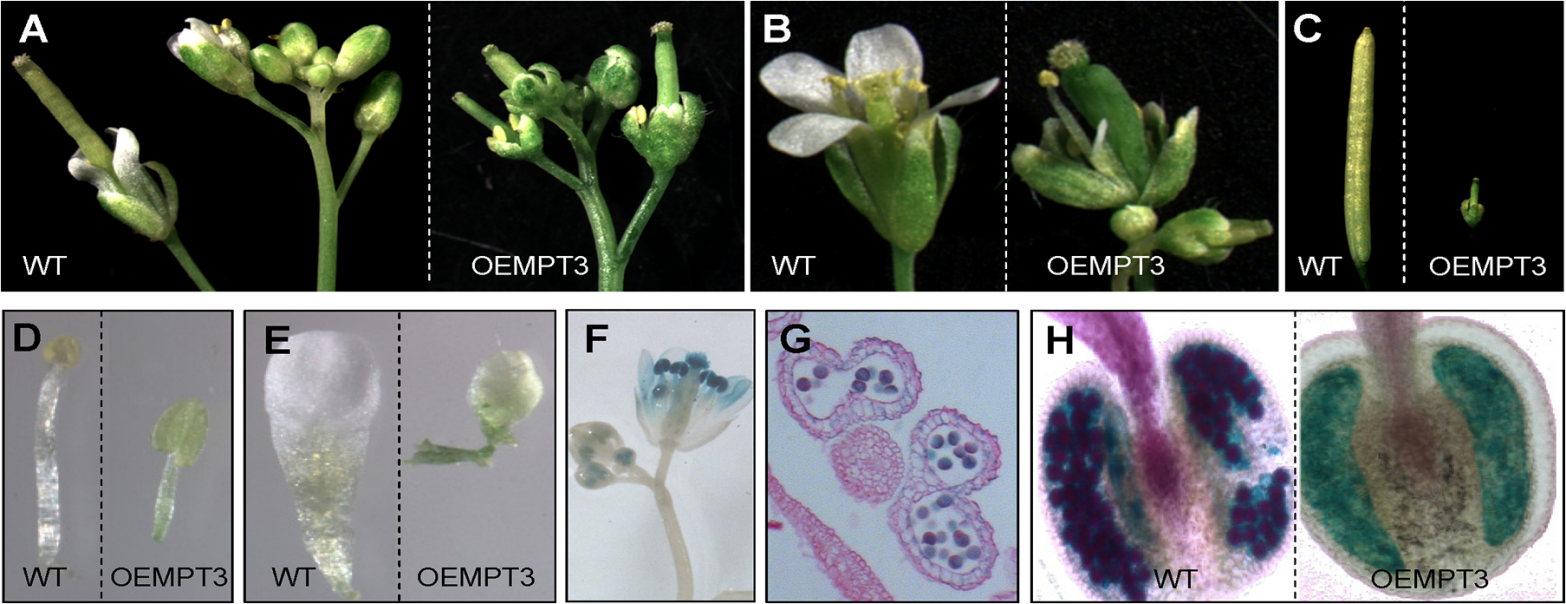
Developmental phenotypes of OEMPT3 plants at reproductive stage. Representative flower (A, B), silique (C), stamen (D) and petal (E) of wild type and OEMPT3 plants at 60 DAP. GUS staining of flower (F) and pollen (G) in OEMPT3 plants. (H) Pollen Alexander staining of wild type and OEMPT3 plants.

At seedling stage, we found no visible differences between the wild type and the OEMPT3 plants under normal growth conditions. However, OEMPT3 plants at 20 DAP (days after planting) exhibited obvious downward-curled leaves with more anthocyanin, dwarfism and growth retardation (Fig 1A, 1B and 1C, S1 Table). The height of OEMPT3 plants was only 1/4 of the wild type plants (Fig 1D and 1E, S1 Table). At reproductive stage, OEMPT3 plants developed pin-like flower with defective sepal, petal, stamen, and consequently reduced fertility (Fig 2A–2E). As *AtMPT3* was highly expressed in pollens (Fig 2F and 2G), we speculated that the reduced fertility was probably due to poor pollen viability. By Alexander staining, we found that the OEMPT3 flowers had almost no viable pollens (Fig 2H). L4 and L14 also exhibited similar developmental defects with L5 (S2 Fig). These results demonstrated that *AtMPT3* overexpression disturbed the development of *Arabidopsis* at both vegetative and reproductive growth stages.

### Genome-wide expression profile analysis of OEMPT3 plants

**Fig 3:**
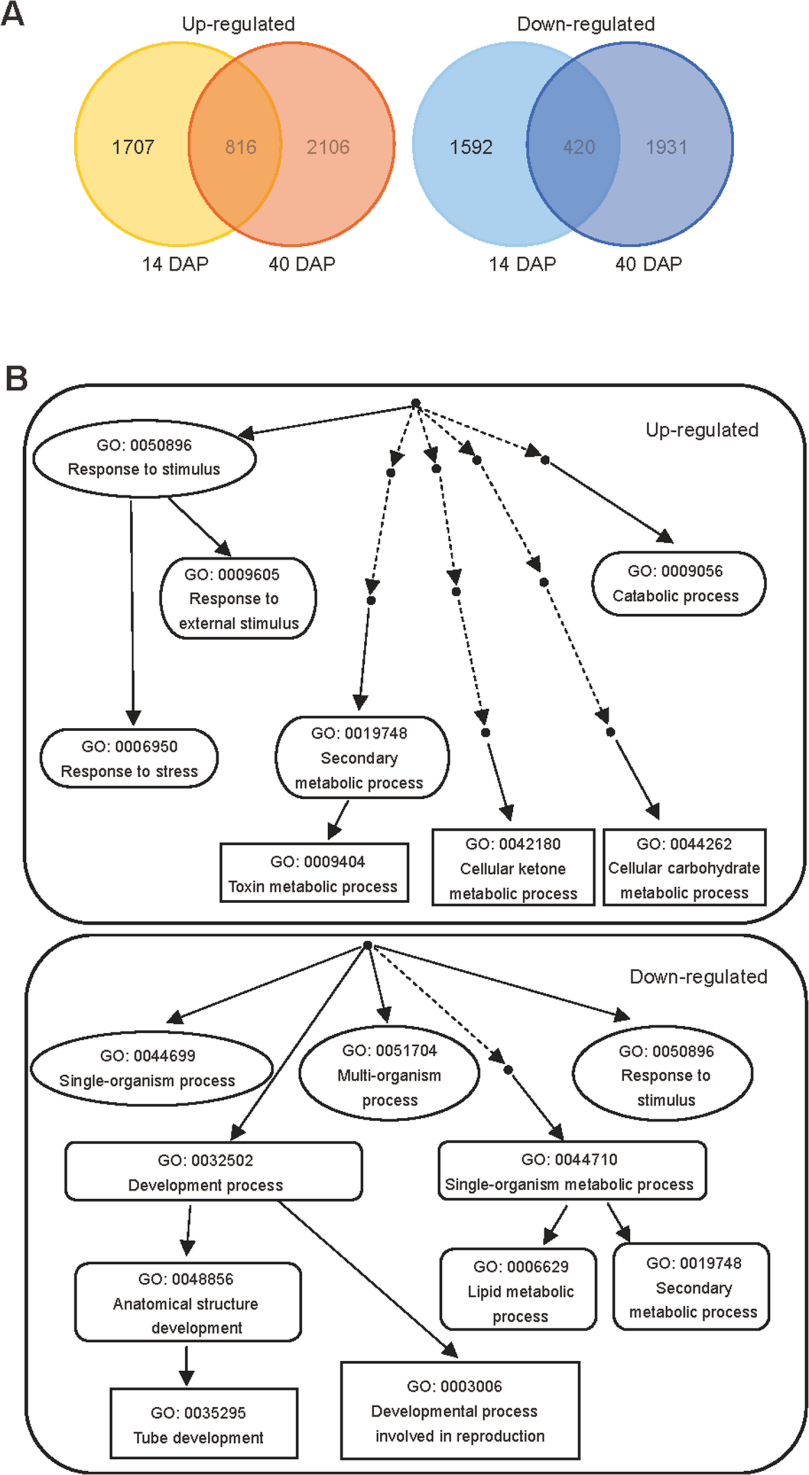
Microarray data analysis of OEMPT3 plants compared to wild type plants. (A) Venn diagram for the proportions of genes affected by *AtMPT3* at 14 and 40 DAP compared to wild type plants. (B) Biological process classification of the up-regulated and down-regulated genes by GOEAST analysis.

**Fig 4:**
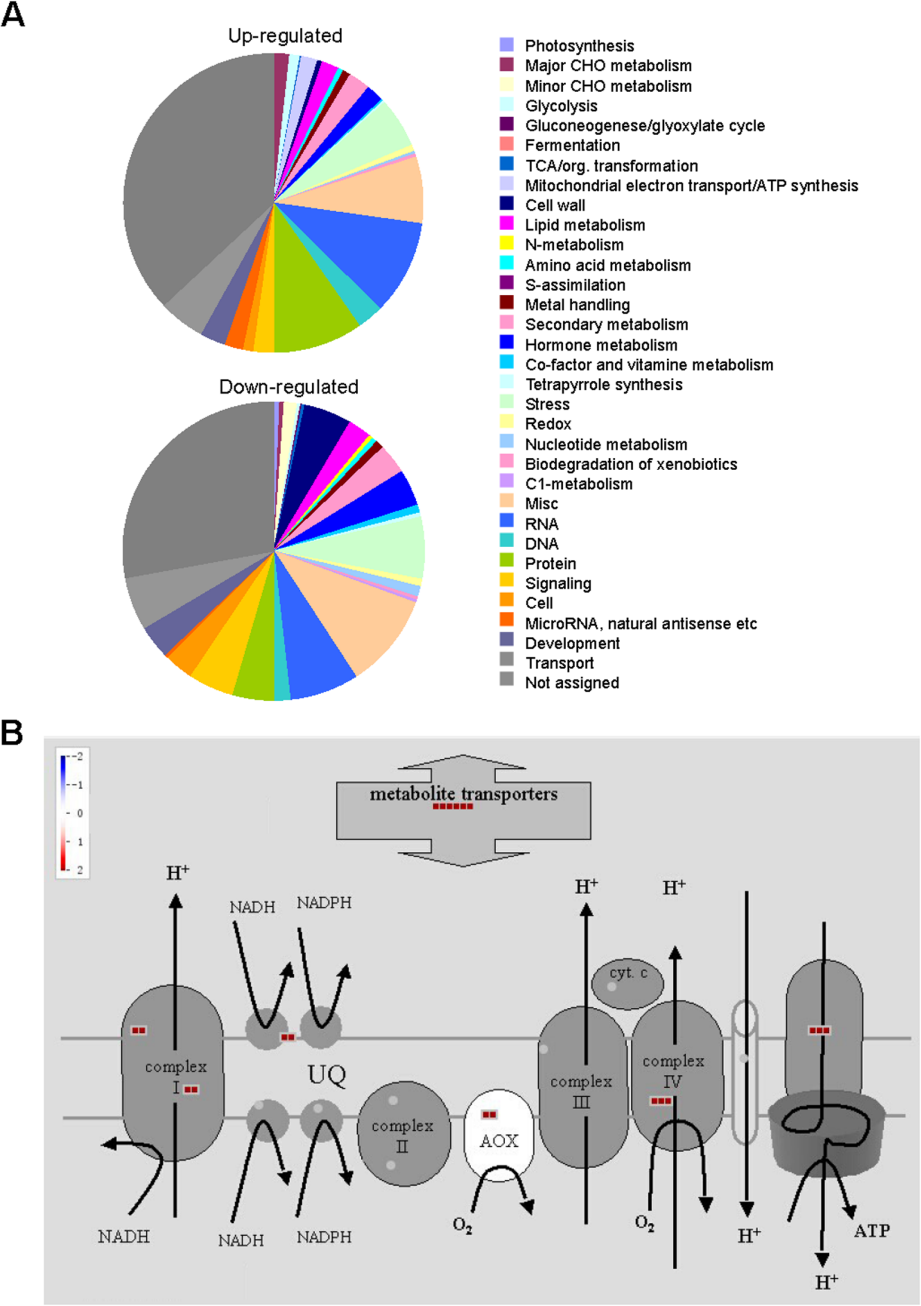
*AtMPT3* regulates a major proportion of genes involved in mitochondrial electron transport. (A) PageMan classification of genes based on their MapMan ontology term allocation. (B) MapMan scheme of genes for the mitochondrial electron transport chain. Red indicates the transcript levels of the genes are increased.

To investigate the mechanisms of *AtMPT3* in modulating plant development, the transcript profiles of wild type and OEMPT3 plants at 14 DAP (no visible difference between wild type and OEMPT3 at this time) and 40 DAP (obvious developmental defects in OEMPT3) were studied by microarray analysis. We found that 1236 genes were differentially expressed at both 14 DAP and 40 DAP OEMPT3 plants compared to wild type plants. As shown in Fig 3A, 816 genes were up-regulated (fold change >2) and 420 genes were repressed (fold change <0.5) in OEMPT3 plants. The biological process classification of the differentially expressed genes by GOEAST (http://omicslab.genetics.ac.cn/GOEAST/index.php) revealed substantial metabolic readjustment in OEMPT3 plants (Fig 3B). The putative function analysis displayed a modified expression of genes involved in stimulus response, cell developmental process and growth, secondary metabolism, cell wall biogenesis, and stamen and pollen growth (Fig 4A). Moreover, genes involved in the respiratory chain (complex I and IV), ATP synthase, alternative oxidases (*AOX*s) and alternative NADH dehydrogenases (*ND*s) were obviously induced in the OEMPT3 plants at both vegetative and reproductive stages (Fig 4B and S2 Table). These results indicated that *AtMPT3* overexpression substantially modulated the transcriptome, especially the genes involved in mitochondrial respiration.

### Overexpression of *AtMPT3* impairs the mitochondrial function in *Arabidopsis*

**Fig 5:**
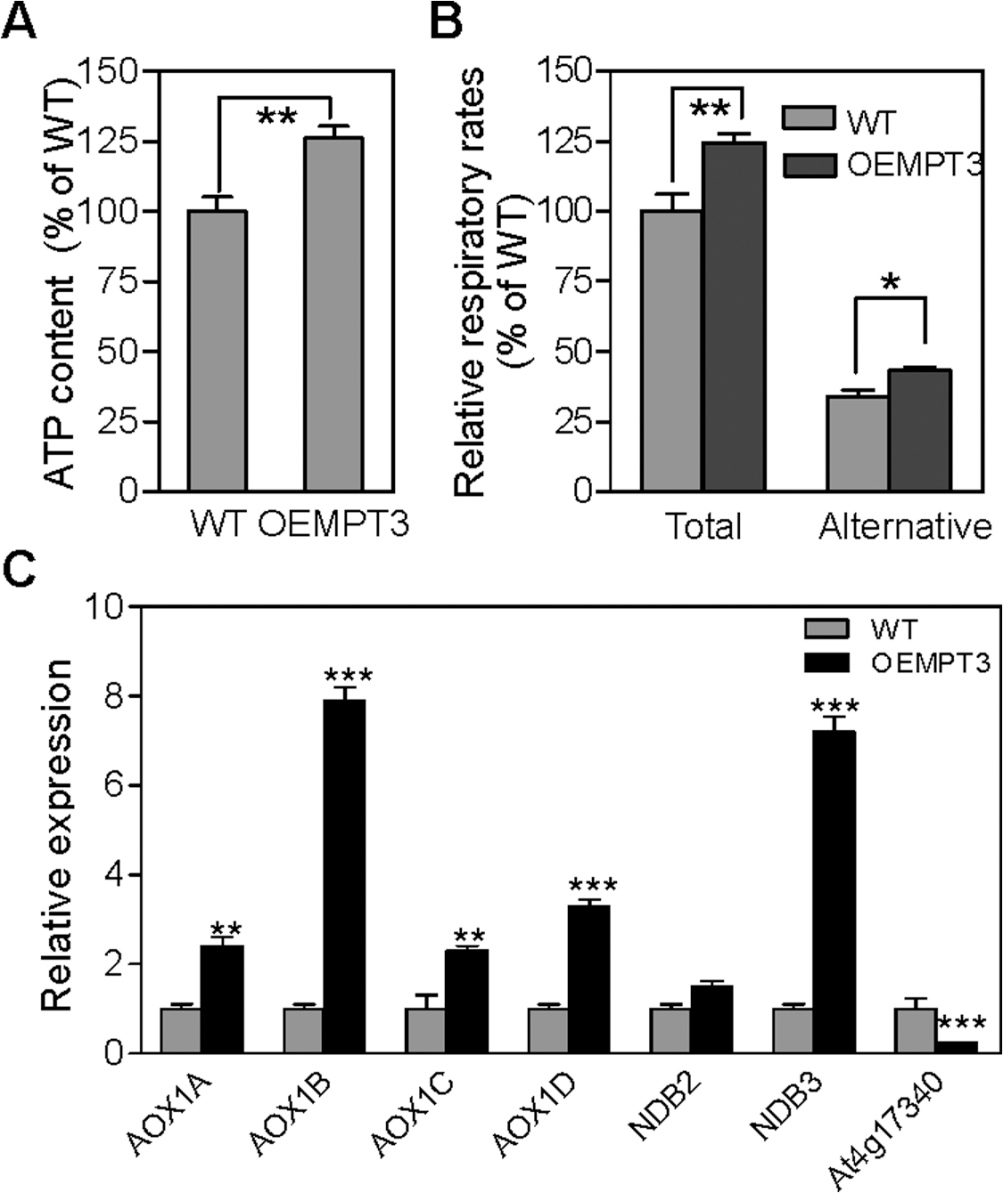
*AtMPT3* impairs mitochondrial function. (A) ATP content and (B) respiration rate of wild type and OEMPT3 plants at 40 DAP. Values represent the average of three replicates. (C) Real time RT-PCR analysis of genes involved in alternative respiration in 40 DAP wild type and OEMPT3 plants. The graphs indicate the induction fold of these genes compared with the control. *P*-value was determined by Student’s *t* test (*P<0.05, **P<0.01, ***P<0.001).

To investigate the possible relationship between the mitochondrial function and *AtMPT3*, we measured the ATP content and the respiration rate in wild type and OEMPT3 plants. As shown in Fig 5A, an increase of approximately 26% ATP content was detected in the OEMPT3 plants under normal conditions. The total respiration rate assay also showed a nearly 1.3-fold increase (Fig 5B), suggesting an enhanced electron transport activity in the mitochondrial inner membrane of OEMPT3 plants, which coincided with the microarray data (Fig 4B and S2 Table) and a higher ATP level (Fig 5A). The alternative respiratory pathway could also be activated when the electron transport chain dysfunctioned in plants [41]. Then we detected a 1.4-fold increase of alternative respiration rate in OEMPT3 plants (Fig 5B). Consistent with the increased alternative respiration, the expression level of *AOX1A*, *AOX1B*, *AOX1C*, *AOX1D*, *NDB2* and *NDB3* were considerably higher in OEMPT3 plants, especially *AOX1B* and *NDB3* (Fig 5C). In addition, the tonoplast intrinsic *TIP2;2* (At4g17340), a marker gene with repressed expression in response to various mitochondrial dysfunctions, was also obviously down-regulated (Fig 5C). Collectively, these results showed that overexpression of *AtMPT3* indeed led to mitochondrial dysfunction in *Arabidopsis*.

### Overexpression of *AtMPT3* induces ROS accumulation

**Fig 6:**
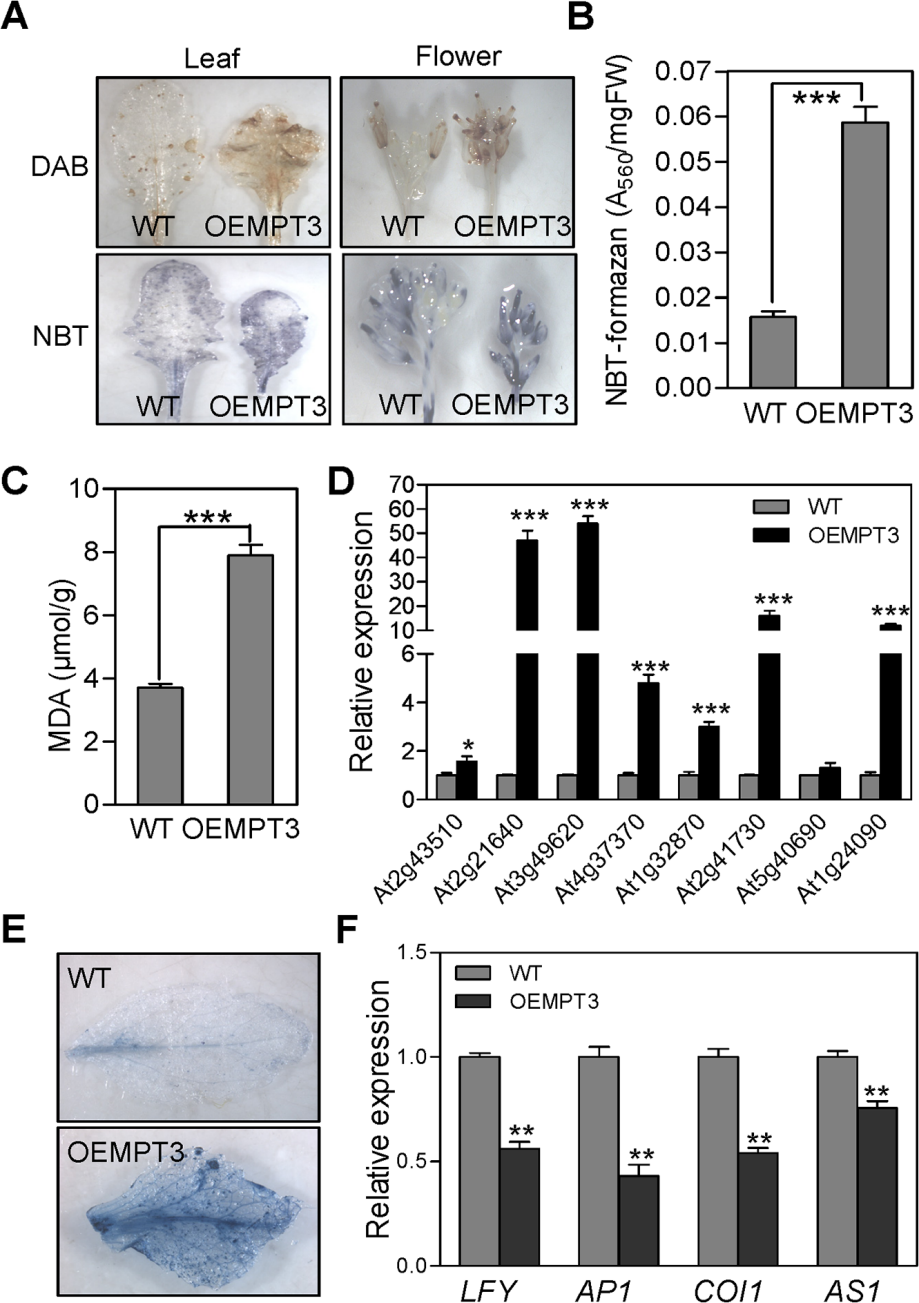
*AtMPT3* alters the cellular redox homeostasis and represses the expression of genes involved in leaf and flower development. (A) ROS histological staining analysis of wild type and OEMPT3 plants. (B) NBT-formazan content analysis and (C) MDA content of wild type and OEMPT3 plants at 40 DAP. Real time RT-PCR analysis of ROS responsive genes (D) and leaf and flower development genes (F) in 40 DAP wild type and OEMPT3 plants. Mean values from three independent replicates are normalized to the levels of an internal control, *GAPDH*. Error bar indicates SD (n = 3). *P*-value was determined by Student’s *t* test (*P<0.05, **P<0.01, ***P<0.001). (E) Trypan blue staining of the leaves of wild type and OEMPT3 plants at 40 DAP.

Besides the photosynthesis process in chloroplasts, the respiration in mitochondrion is another major source of ROS production [42]. Given the disturbed mitochondrial respiration process, we presumed that oxidative stress might occur in OEMPT3 plants. To test our hypothesis, we examined the ROS content in wild type and OEMPT3 plants. As shown in Fig 6A, DAB staining indicated that both the leaves and flowers of OEMPT3 plants accumulated higher amounts of H_2_O_2_ than wild type plants. Similarly, qualitative and quantitative analysis all revealed a higher O_2_^-^ level in OEMPT3 plants (Fig 6A and 6B). In order to exclude the possibility that the increased ROS content was caused by chloroplasts, the photosynthetic parameters of wild type and OEMPT3 plants was detected under normal growth conditions. As shown in S3 Table, the photosynthetic parameters, including Fv/Fm and O_2_ evolution, were indistinguishable between the wild type and OEMPT3 plants under the same growth conditions, suggesting that the chloroplasts of OEMPT3 plants were intact and mitochondria would be the major source of excess ROS in OEMPT3 plants. In addition, the OEMPT3 plants accumulated more MDA (malondialdehyde) and transcripts of ROS responsive genes (Fig 6C and 6D). As a result, much severer PCD (programmed cell death) occurred in OEMPT3 plants (Fig 6E). Similarly, L4 and L14 plants also showed higher ATP content, faster respiration rate and more ROS compared with wild type plants (S3 Fig). Taken together, these results suggested that overexpression of *AtMPT3* disturbed the cellular redox homeostasis and gave rise to ROS accumulation in transgenic *Arabidopsis*, which was associated with the defects in flower development and male gametogenesis.

### The expression of leaf and flower development associated genes are repressed in OEMPT3 plants

To further explore the mechanisms of *AtMPT3* in regulating development, we examined the expression levels of major genes involved in leaf and flower development by real time PCR analysis. The results showed that the expression of these genes, *LFY* (*LEAFY*), *AP1* (*APETALA1*), *COI1* (*CORONATINE INSENSITIVE 1*), *AS1* (*ASYMMETRIC LEAVES 1*), were comparable between the wild type and the OEMPT3 plants at 14 DAP (data not shown). However, when reaching the reproductive growth stage (40 DAP), these genes were apparently suppressed in OEMPT3 plants (Fig 6F), which was in accordance with the transcript profile analysis (S4 Table). These results revealed that in addition to the ROS overaccumulation, the decreased transcription level of *LFY*, *AP1*, *COI1* and *AS1* also contributed to the defective development of leaf and flower in OEMPT3 plants.

## Discussion

Mitochondria play vital roles in diverse cellular metabolisms. To date, increasing reports have proved that the altered expression of mitochondrion-associated genes would affect both plant growth and development [4, 6]. The concerted function of intramitochondrial and extramitochondrial metabolism relied on the finely tuned transport of various metabolites, nucleotides, and cofactors across the inner mitochondrial membrane, which was mainly realized by the mitochondrial carrier family proteins [9]. Biochemical characterization of plant mitochondrial carrier function over the last 40 years has revealed the operation of carriers for phosphate, adenine nucleotides, mono-, di-, and tri-carboxylates, amino acids, and cofactors such as NAD^+^ and coenzyme A [9, 43]. Among them, the MPT, which located in the mitochondrial inner membrane, could catalyze the phosphate (H_2_PO_4_^-^)/proton symport, the phosphate/hydroxyl ion antiport, and the exchange of mitochondrial matrix with cytosolic phosphate [21]. In this study, we provide evidence that overexpression of *AtMPT3* exhibited multiple morphological and physiological changes, such as curly leaf, abnormal flower, dwarfism, higher ATP content and respiration rate, which was due to mitochondrial dysfunction (Figs 1, 2 and 5). To test whether the development defects were caused by the misregulation of other mitochondrial carrier genes, we analyzed the expression levels of *AtMPT1*, *AtMPT2* and other three members in OEMPT3 plants, including adenine nucleotide translocator (At3g08580 and At5g13490) and dicarboxylate/tricarboxylate carrier (At5g19760). The results showed that the mRNA levels of these genes were comparable in wild type and OEMPT3 plants, indicating that *AtMPT3* did not affect the expression of other mitochondrial carrier (S4 Fig). In addition, we examined the T-DNA insertion mutant for *AtMPT3* (SALK_010017). However, only heterozygous mutant (no visible developmental defect) could be obtained, suggesting that complete loss of *AtMPT3* was lethal and *AtMPT3* was indispensable for *Arabidopsis* development. We also cloned the other two *MPT*s (*AtMPT1* and *AtMPT2*) from *Arabidopsis*, and obtained respective overexpression lines. Unlike *AtMPT3*, no apparent difference was observed in these transgenic lines (S5 Fig). We suggested that *AtMPT3* was more effective and important in regulating plant growth and development compared with *AtMPT1* and *AtMPT2*.

Mitochondrial dysfunction usually brings about the excessive generation of ROS, which are well-known signaling molecules in diverse processes [42]. ROS content was closely correlated with plant growth and development, and its accumulation usually resulted in vegetative and reproductive growth defects [25, 44, 45]. Consistent with this, the OEMPT3 plants accumulated higher H_2_O_2_ and O_2_^-^ levels and concomitantly severer PCD than wild type plants under normal conditions (Fig 6A–6E), which accounted for the developmental phenotypes. Moreover, gene chip analysis showed that numerous genes involved in multiple metabolic pathways including cell development and growth, secondary metabolism, mitochondrion electron transport, and stamen and pollen growth were significantly readjusted in OEMPT3 plants (Figs 3B and 4A). In accordance with the microarray data, real time RT-PCR analysis detected obvious activation of genes involved in cellular redox homeostasis and alternative respiration pathway (Figs 5C and 6D), and remarkably suppression of genes participated in plant growth and development in OEMPT3 plants (Fig 6F), respectively. Besides the experimentally validated aspects, genes involved in other processes, such as response to external stimulus and hormone metabolism (Figs 3B and 4A), may also correlated with the phenotypes of OEMPT3 plants. Taken together, we concluded that the oxidative intracellular status, modified transcription and metabolism in OEMPT3 plants synergistically gave rise to the developmental abnormalities.

This study indicated that *AtMPT3* played important roles in regulating plant growth and development in *Arabidopsis*. Based on known data, we proposed that overexpression of *AtMPT3* elevated the Pi concentration in mitochondrial matrix, which accelerated the subsequent processes of electron transport, ATP biosynthesis, ROS accumulation and PCD. In addition, the nucleus received the retrograde signal from mitochondria and reset the entire expression profile accordingly. Then, the final developmental characteristics of OEMPT3 plants were generated. Although this research presented useful clues of *AtMPT3* in regulating development, further studies, such as the relationship between *AtMPT3* and oxidative phosphorylation, the upstream events suppressing *LFY*, *AP1*, *COI1* and *AS1*, the retrograde signal triggered by mitochondrial dysfunction, are worthy to be conducted to explain the details in the future.

## Supporting Information

S1 Fig Expression levels of *AtMPT3* in 2-week-old wild type and transgenic plants. (TIF)

S2 Fig Phenotypes of *AtMPT3* overexpression plants from L4 and L14. (A, B) Phenotypes of L4 and L14 overexpression plants compared to wild type at 30 DAP. (C, D, E) Phenotypes of L4 and L14 overexpression plants compared to wild type at 60 DAP. Representative leaf (F, G) and flower (H, I) of wild type and L4 and L14 overexpression plants at 60 DAP.
(TIF)

S3 Fig ATP content (A), respiration rate (B) and ROS histological staining analysis (C) of wild type and transgenic plants from L4 and L14. (TIF)

S4 Fig Expression levels of mitochondrial carriers in OEMPT3 plants. (TIF)

S5 Fig Phenotype comparison of wild type and *AtMPT1*, *AtMPT2*, *AtMPT3* overexpression plants. (TIF)

S1 Table Comparison of wild type and OEMPT3 plants (*P<0.05, **P<0.01). (DOC)

S2 Table List of differentially expressed genes (> 2 fold increase and *P* < 0.05) involved in mitochondrial electron transport in OEMPT3 plants. (DOC)

S3 Table Photosynthetic parameters of wild type and OEMPT3 plants under normal growth conditions (*P<0.05, **P<0.01, ***P<0.001). (DOC)

S4 Table Transcription levels of *LFY*, *AP1*, *COI1* and *AS1* in genome-wide expression profile analysis (OEMPT3 plants compared to wild type plants, *P* < 0.05). (DOC)

S5 Table Primers used in this study. (DOC)
