# Supplementary figures and images for: Overexpression of Mitochondrial Phosphate Transporter 3 Severely Hampers Plant Development through Regulating Mitochondrial Function in Arabidopsis
Source: PLoS One. 2015 Jun 15;10(6):e0129717. doi: 10.1371/journal.pone.0129717 (PMC4468087; doi:10.1371/journal.pone.0129717)

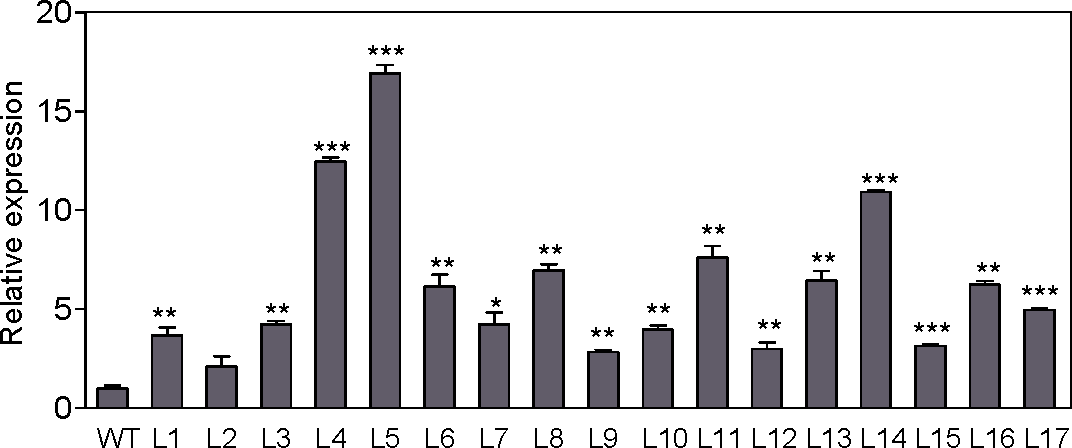

Supplement: S1 Fig — (TIF) [file pone.0129717.s001.tif]

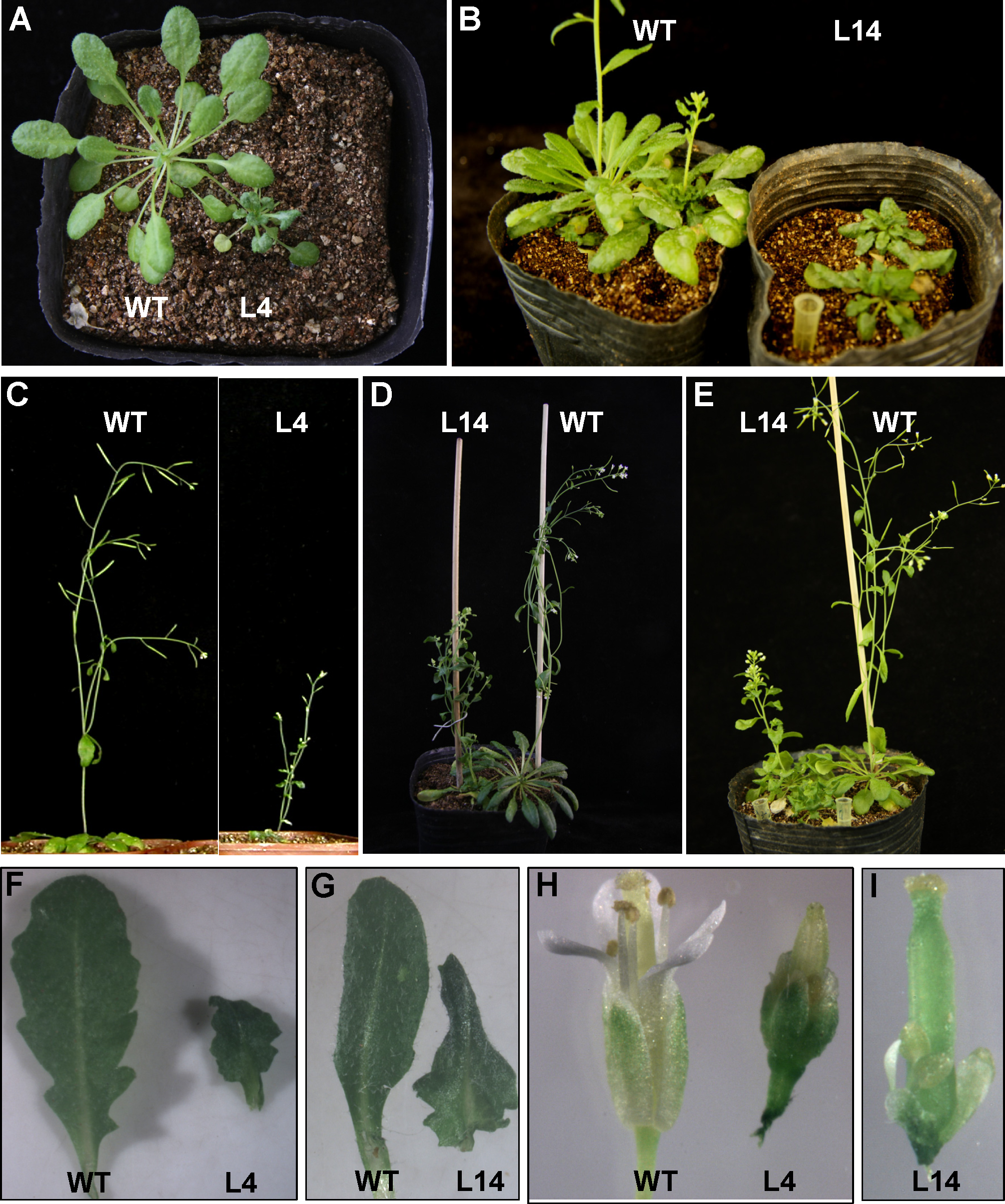

Supplement: S2 Fig — (A, B) Phenotypes of L4 and L14 overexpression plants compared to wild type at 30 DAP. (C, D, E) Phenotypes of L4 and L14 overexpression plants compared to wild type at 60 DAP. Representative leaf (F, G) and flower (H, I) of wild type and L4 and L14 overexpression plants at 60 DAP. (TIF) [file pone.0129717.s002.tif]

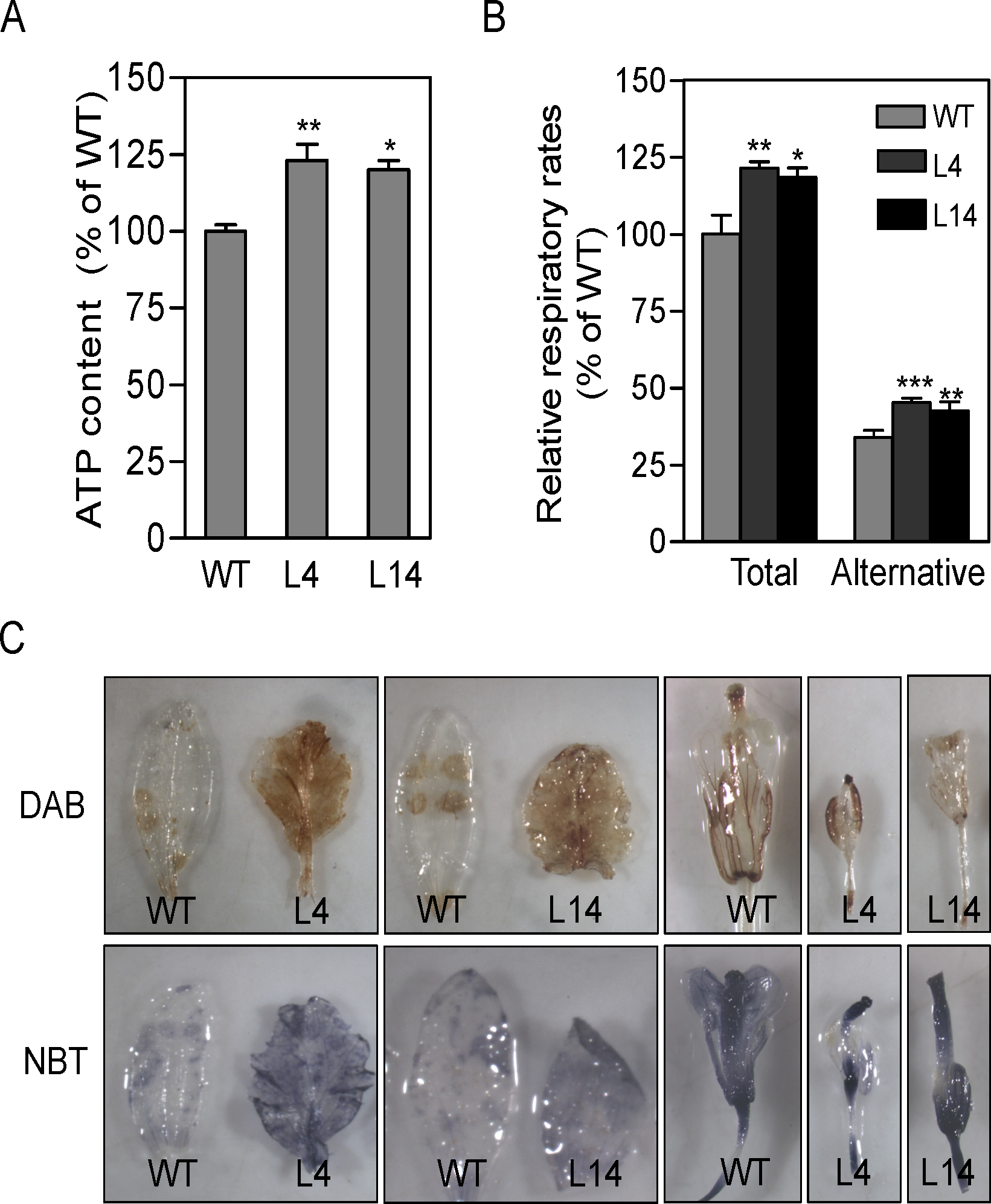

Supplement: S3 Fig — (TIF) [file pone.0129717.s003.tif]

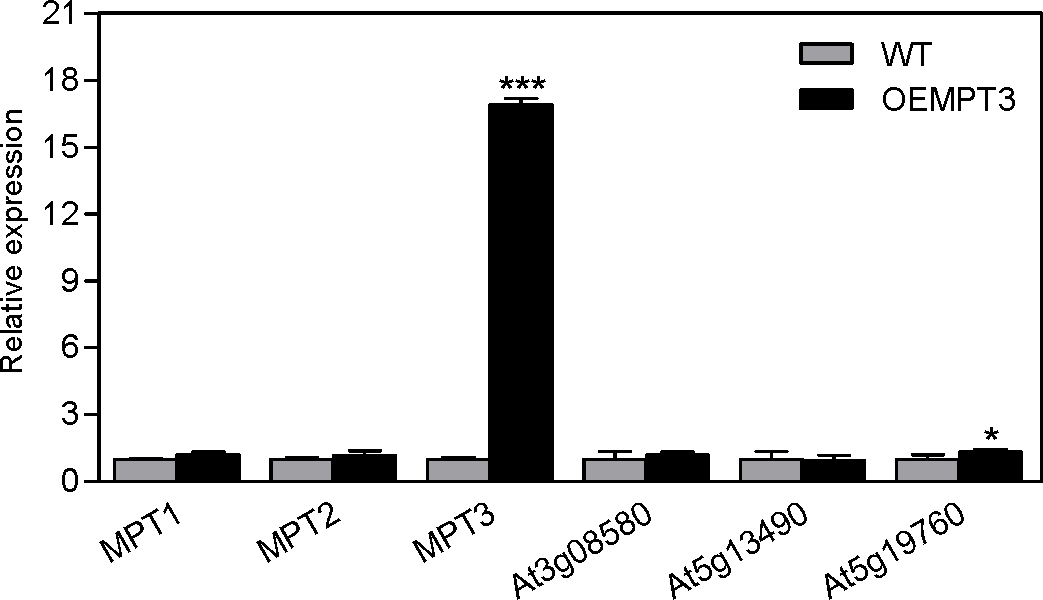

Supplement: S4 Fig — (TIF) [file pone.0129717.s004.tif]

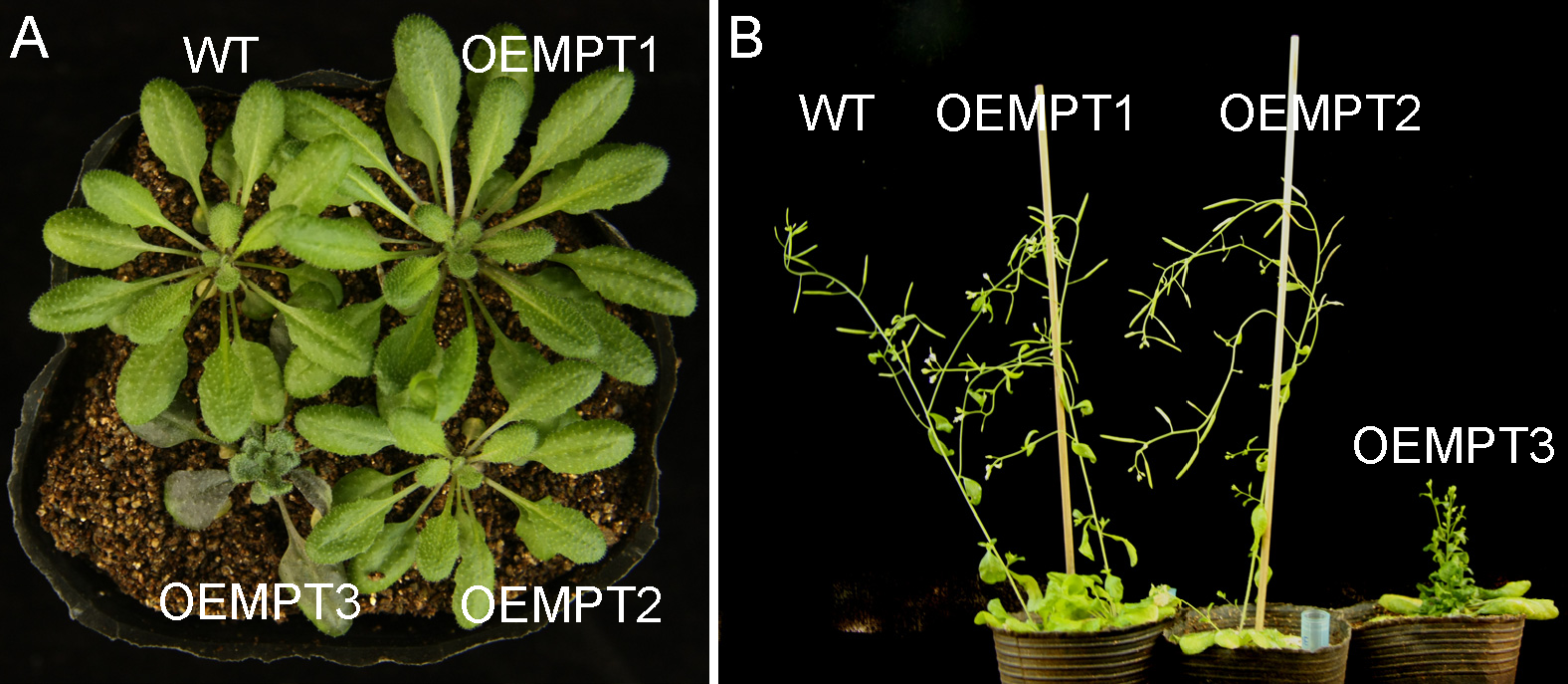

Supplement: S5 Fig — (TIF) [file pone.0129717.s005.tif]
