# Supplementary material for: Overexpression of Mitochondrial Phosphate Transporter 3 Severely Hampers Plant Development through Regulating Mitochondrial Function in Arabidopsis
Source: PLoS One. 2015 Jun 15;10(6):e0129717. doi: 10.1371/journal.pone.0129717 (PMC4468087; doi:10.1371/journal.pone.0129717)
Supplement: S1 Table — (DOC) [file pone.0129717.s006.doc]

|  | Anthocyanin  content  [(A_535_-A_650_) g^-1^ FW] | Height of stem (cm) | Radius of rosette leaves (cm) | Radius of cauline leaves (cm) |
| --- | --- | --- | --- | --- |
| WT | 0.68±0.12 | 31.2±3.4 | 1.3±0.3 | 1.2±0.2 |
| OEMPT3 | 2.42±0.45  * | 7.5±1.3  ** | 0.8±0.2  * | 0.8±0.1 |

**Table S1 Comparison of wild type and OEMPT3 plants** (**P*<0.05, ***P*<0.01)**.**
