# Supplementary material for: Overexpression of Mitochondrial Phosphate Transporter 3 Severely Hampers Plant Development through Regulating Mitochondrial Function in Arabidopsis
Source: PLoS One. 2015 Jun 15;10(6):e0129717. doi: 10.1371/journal.pone.0129717 (PMC4468087; doi:10.1371/journal.pone.0129717)
Supplement: S2 Table — (DOC) [file pone.0129717.s007.doc]

**Table S2 List of differentially expressed** **genes ( > 2 fold increase and *P* < 0.05 ) involved in mitochondrial electron transport in OEMPT3 plants.**

| AGI | 14 DAP | 40 DAP | Description |
| --- | --- | --- | --- |
| ATMG00160 | 2.873586 | 2.131399 | Cytochrome c oxidase subunit 2 |
| ATMG01360 | 2.119937 | 2.157168 | Cytochrome c oxidase subunit 1 |
| ATMG00060 | 2.381699 | 2.343056 | Mitochondrial NADH dehydrogenase subunit 5 |
| ATMG01170 | 2.041093 | 2.420936 | ATPase subunit 6 |
| ATMG00990 | 3.269373 | 2.594034 | NADH dehydrogenase subunit 3 |
| ATMG01275 | 4.323266 | 2.733601 | Subunit of mitochondrial NAD(P)H dehydrogenase |
| AT1G18320 | 3.056813 | 2.85244 | Mitochondrial import inner membrane translocase subunit Tim17/Tim22/Tim23 family protein |
| AT4G27940 | 2.091734 | 2.978695 | Mitochondrial substrate carrier family protein |
| AT5G14040 | 4.158449 | 2.983255 | Mitochondrial phosphate transporter |
| AT2G07687 | 2.40804 | 2.990137 | Cytochrome c oxidase subunit 3 |
| AT2G37890 | 2.782645 | 3.074427 | Mitochondrial substrate carrier family protein |
| AT3G27620 | 4.002191 | 5.158567 | AOX1C (alternative oxidase 1C); alternative oxidase |
| AT5G09470 | 21.04052 | 5.215137 | Mitochondrial substrate carrier family protein |
| AT1G64220 | 4.769738 | 6.175792 | Translocase of outer membrane 7 kDa subunit 2 (TOM7-2) |
| AT5G60730 | 4.01424 | 6.240377 | Anion-transporting ATPase family protein |
| AT3G22370 | 6.720353 | 7.428938 | AOX1A (alternative oxidase 1A); alternative oxidase |
| AT5G44140 | 2.856695 | 9.677814 | ATPHB7 (PROHIBITIN 7) |
| AT2G20800 | 70.11359 | 193.0829 | NDB4; NADH dehydrogenase |
| AT4G21490 | 33.30778 | 29.95523 | NDB3; NADH dehydrogenase |
| AT2G07698 | 2.047544 | 2.011574 | ATP synthase alpha chain, mitochondrial, putative |
