# Supplementary material for: Overexpression of Mitochondrial Phosphate Transporter 3 Severely Hampers Plant Development through Regulating Mitochondrial Function in Arabidopsis
Source: PLoS One. 2015 Jun 15;10(6):e0129717. doi: 10.1371/journal.pone.0129717 (PMC4468087; doi:10.1371/journal.pone.0129717)
Supplement: S3 Table — (DOC) [file pone.0129717.s008.doc]

**Table S3 Photosynthetic parameters of wild type and OEMPT3 plants under normal growth conditions** (**P*<0.05, ***P*<0.01, ****P*<0.001)**.**

|  | Fv/Fm [Leaf number (from top)] | | | | O_2_ evolution  (nmol/ml/min/g FW) |
| --- | --- | --- | --- | --- | --- |
|  | 5 | 6 | 7 | 8 |  |
| WT | 0.823±0.01 | 0.842±0.02 | 0.833±0.01 | 0.835±0.02 | 419.23±18.98 |
| OEMPT3 | 0.831±0.03  ** | 0.826±0.02  *** | 0.834±0.03  * | 0.824±0.01  ** | 404.82±14.23  ** |
