# Supplementary material for: Overexpression of Mitochondrial Phosphate Transporter 3 Severely Hampers Plant Development through Regulating Mitochondrial Function in Arabidopsis
Source: PLoS One. 2015 Jun 15;10(6):e0129717. doi: 10.1371/journal.pone.0129717 (PMC4468087; doi:10.1371/journal.pone.0129717)
Supplement: S4 Table — (DOC) [file pone.0129717.s009.doc]

| Gene | 14 DAP | 40 DAP | Description |
| --- | --- | --- | --- |
| *LFY* | 1.036729 | 0.455304 | *LEAFY*; transcription factor that promotes the transition to flowering |
| *AP1* | 1.196024 | 0.325552 | *APETALA1*; DNA binding / transcription factor, Floral homeotic gene encoding a MADS domain protein |
| *COI1* | 1.113661 | 0.343056 | *CORONATINE INSENSITIVE 1*, SCF ubiquitin ligase complex |
| *AS1* | 1.201914 | 0.777486 | *ASYMMETRIC LEAVES 1*, MYB-domain protein involved in specification of the leaf proximodistal axis |

Table S4 Transcription levels of *LFY*, *AP1*, *COI1* and *AS1* in genome-wide expression profile analysis (OEMPT3 plants compared to wild type plants, *P* < 0.05).
