# Supplementary material for: Overexpression of Mitochondrial Phosphate Transporter 3 Severely Hampers Plant Development through Regulating Mitochondrial Function in Arabidopsis
Source: PLoS One. 2015 Jun 15;10(6):e0129717. doi: 10.1371/journal.pone.0129717 (PMC4468087; doi:10.1371/journal.pone.0129717)
Supplement: S5 Table — (DOC) [file pone.0129717.s010.doc]

**Table S4 Primers used in this study**.

| Primer name | sequence | Primer name | sequence |
| --- | --- | --- | --- |
| AtMPT3-F | GGATCCATGGAATCTCCGAAGAATTCTCTG | AT4G37370-F | TTCTCCAATCACCGTCTCAATAGC |
| AtMPT3-R | GAGCTCTTAGGCTTTGGCTTCAGTAGCT | AT4G37370-R | CTCAACACCGTCTCCGTAGTAAC |
| MPT3P-F | CTGCAGTAGCTTCCTTAGACGGTTGACAC | AT1G32870-F | GTTCTGGTCTCCTGAATGATGATG |
| MPT3P-R | GGATCCGGCGGCGGCGGAGGAAGGA | AT1G32870-R | GCTTGGTTCTTCCCTGATTGC |
| QMPT1-F | TGTCTTGCTGGCTATACTG | AT2G41730-F | CGTCACCAAGGCATCGTAAG |
| QMPT1-R | CATTCTTGGCTTTGTTGTTG | AT2G41730-R | TGGACCGCTCGCTTTCTC |
| QMPT2-F | CGATTACGCCACTTGATG | AT5G40690-F | TGCCTCCGTCGCTGAATC |
| QMPT2-R | GCTCTTTGATTGTTGTCTTG | AT5G40690-R | ACCTTGCTTGACCACATTTCC |
| QMPT3-F | AAAGAGCAAGGAGTCAAAGG | AT1G24090-F | GTACTCGCAAAGCTCCACAAG |
| QMPT3-R | CAGCAAGGTCAGAGTAAGTC | AT1G24090-R | TCACGCCACCTCAACTTCTC |
| AOX1A-F | CTTCTGATTCTCGTCCTCCT | AT4G17340-F | GTCTCCTCCTCGTCTTCGTTAC |
| AOX1A-R | GACGGTCCGTACGGTTTCG | AT4G17340-R | TCACCACAATCTCCATCACTACTC |
| AOX1B-F | TCTTTTAGCTCACGACCTTGG | LFY-F | CCACTGCCTAGACGAAGAAG |
| AOX1B-R | TGGAGACTTGAAGCTGATGC | LFY-R | GACGACAAGCGATGTTCAC |
| AOX1C-F | CGGCTATCGCTGTTGATTAC | AP1-F | ATTCTTAGGGCTCAACAG |
| AOX1C-R | CCTTGGTAGTGAATATCGGATG | AP1-R | ATCATTCCTCCTCATTGC |
| AOX1D-F | ACCCAAACTTGCTCATCGTATC | COI1-F | CTTCCGCCTTGTCTTACTC |
| AOX1D-R | GCTCGTATAACATAAACCACATCC | COI1-R | CAGCAGCATCCATCTCAC |
| NDB2-F | AATCGGCTCTATCTCAGGTTGAC | AS1-F | CATTATCGCCTTCCACAG |
| NDB2-R | TTCTGGACTCTTCTCACACTCTTC | AS1-R | CTCTACAACACTCCACAAG |
| NDB3-F | CCATTAGGATTAGAGGAGAAGG | AT3G08580-F | CTACCGTGGTCTGTACTTTGGAC |
| NDB3-R | GAGTACCACAGCCATTGAC | AT3G08580-R | CCGTACTTCTTACCGAAGACAAT |
| AT2G43510-F | AAGAATACGGAGGTGATGTTG | AT5G13490-F | GTATCATTGTCTACCGTGGTC |
| AT2G43510-R | CGCAGAAGTCGCATAAGC | AT5G13490-R | AACTGCAGCTTGTCGTAGCC |
| AT2G21640-F | AGGATGATAACGCCACTTTC | AT5G19760-F | GAACATGGGAATGCTTGCATC |
| AT2G21640-R | TGCTGTCTGAAGAGGAGAG | AT5G19760-R | CAGTAAACTGGGAAACCCGAGT |
| AT3G49620-F | CAGACTTCAAATCCCTACTACC | GAPDH-F | TTGGTGACAACAGGTCAAGCA |
| AT3G49620-R | TCCGACTACTTCCACAACC | GAPDH-R | AAACTTGTCGCTCAATGCAATC |
